# Supplementary figures and images for: Urban centre green metrics in Great Britain: A geospatial and socioecological study
Source: PLoS One. 2022 Nov 23;17(11):e0276962. doi: 10.1371/journal.pone.0276962 (PMC9683550; doi:10.1371/journal.pone.0276962)

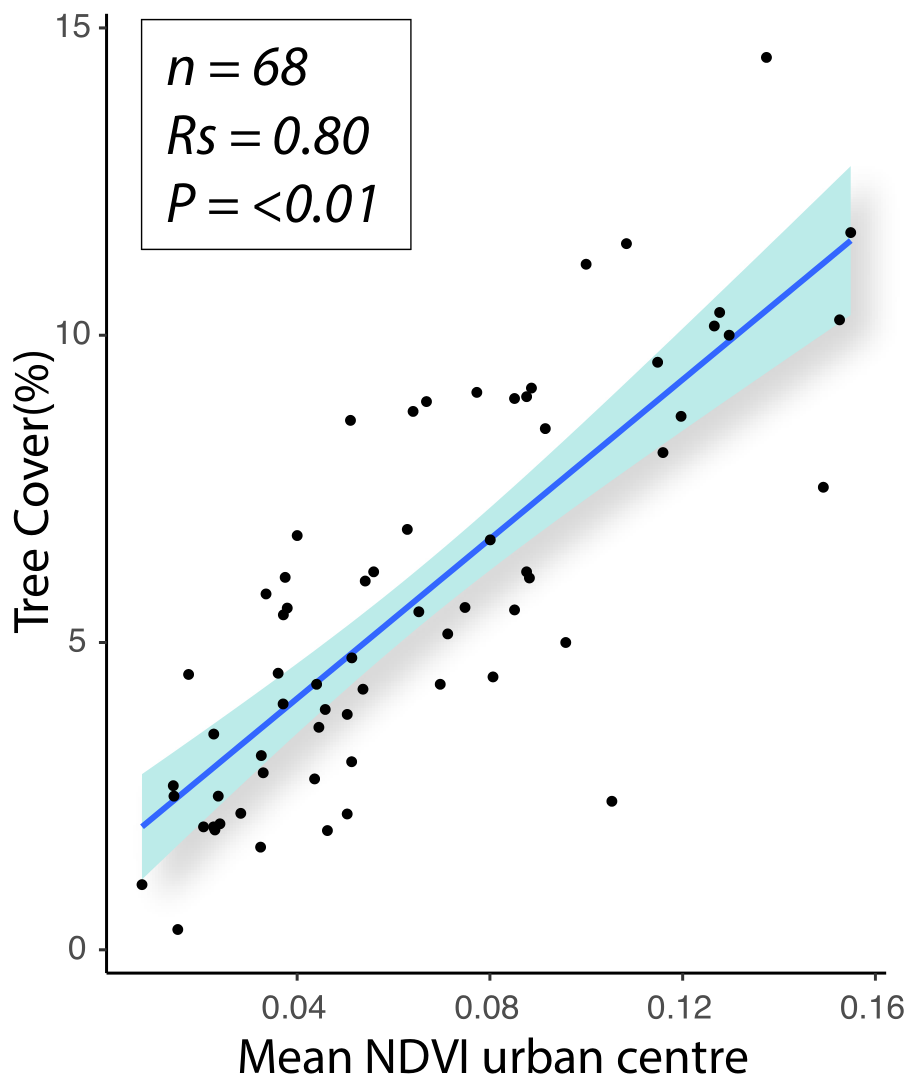

Supplement: S1 Fig — (TIF) [file pone.0276962.s001.tif]
