# Supplementary material for: Urban centre green metrics in Great Britain: A geospatial and socioecological study
Source: PLoS One. 2022 Nov 23;17(11):e0276962. doi: 10.1371/journal.pone.0276962 (PMC9683550; doi:10.1371/journal.pone.0276962)
Supplement: S1 Table — (PDF) [file pone.0276962.s002.pdf]

S1_Table. List of urban centres and their greenspaces as defined by the OS Greenspace (OSGS) dataset. The table also contains the area (in m^2) of greenspace for each urban centre.

Urban centre name	Community growing space	Bowling Green	Cemetery	Play Space	Playing Field	Public Park Or Garden	Religious Grounds	Tennis Court	Total	Total OSGS area (sq. metres)
Aberdeen			1			2			3	11085
Basildon							1		1	2484
Basingstoke						2	1		3	14108
Birmingham Central							3		3	25462
Bournemouth						4	2		6	20100
Brighton				2		3	1		6	21929
Bristol Central			1			5	3		9	36325
Bromley						2	1		3	12733
Cambridge				1		2	5	2	10	8496
Camden Town				4		3			7	6184
Cardiff Central		1			1	2	1		5	5941
Chelmsford				1		1	2		4	16679
Chelsea			1	2		1			4	20495
Cheltenham		1		1		5	2		9	14378
City of London		1	4	10	1	14	2		32	38116
Colchester				1		1	3		5	7164
Coventry						2	2		4	12988
Crawley				1		1	2		4	14976
Croydon						1	3		4	3709
Derby	1					1	3		5	9873
Dundee			1			1	2		4	19026
Ealing						2	1		3	9838
Eastbourne							1		1	7
Edinburgh Central						3	2		5	3395
Exeter			2			2	2		6	34168
Falkirk			1				1		2	1423
Glasgow Central						1	1		2	1420
High Wycombe							1		1	5937
Huddersfield						1			1	5057
Hull			1			1			2	9613
Ipswich			1	1		1	2		5	7356
Islington				6		9			15	20856
Kingston						1	1		2	2856
Leeds Central			1	1		2	1		5	4748
Leicester							4		4	13284
Lincoln			2			1	2		5	6632
Liverpool Central						1	1		2	15746
Maidstone			1			2	1		4	4837
Manchester Central						3	1		4	12086
Marylebone			1	13		28	5	1	48	100353
Middlesbrough						1			1	3855
Milton Keynes				1		1			2	13415
Motherwell										0

Newcastle Upon Tyne							4		4	11943
Northampton			1			1	3		5	9249
Norwich				2		6	13		21	43379
Nottingham Central						3	4		7	16461
Oxford							4		4	5339
Peterborough					1		2		3	2108
Plymouth			1	2		3	1		7	2496
Reading				1			4		5	12291
Richmond Upon Thames			1			1	1		3	5686
Romford							2		2	5270
Sheffield						3	1		4	13330
Shepherd's Bush				2		1			3	10087
Solihull		1				1	1		3	2054
Southampton						2			2	2541
Stockport							1		1	6185
Stoke-On-Trent						1	1		2	2543
Sunderland						1	1		2	3123
Sutton Coldfield							1		1	4025
Swansea			1	1		1	2		5	10579
Watford						1	1		2	7209
Wigan						2			2	4714
Woking										0
Worcester						1	1		2	3375
Worthing						2			2	5282
York		1	2			5	7		15	20937
**Grand Total**	**1**	**5**	**24**	**53**	**3**	**148**	**121**	**3**	**358**	809339

4

5
