# Supplementary material for: Urban centre green metrics in Great Britain: A geospatial and socioecological study
Source: PLoS One. 2022 Nov 23;17(11):e0276962. doi: 10.1371/journal.pone.0276962 (PMC9683550; doi:10.1371/journal.pone.0276962)
Supplement: Attachment — Submitted filename: Review PONE-D-21-34255_reviewer.docx [file pone.0276962.s004.docx]

I have reviewed the manuscript titled "Urban Centre Greenness, Tree Cover, and Green Spaces in Great Britain: A Geospatial Study" and I do find it suitable for the **PLOS ONE** but with the **Major revision required**. I have following observations on this MS.

1. The MS does contribute new in terms of methodology – but a set of well-known methods are available to apply on the research area and their physical parameters. I fail to see any fruitful discussion on the generated datasets. The scientific problem is analyzed and solved. The introduction part is clear and the scientific problem has clearly identified and addressed.
2. The authors discuss clear point about the Greenness tree cover but not emphasize on application - they have studied Great Britain area and their MS used the Geospatial application.
3. The introduction is good, the method section is trivial and vague at places. Discussion is existing but not extensive. Increase the literature and cite in the introduction part (more than 70 papers cited which is more enough but I find many papers not related to the study work. Please remove extra cited papers and be relevant to the research topic).
4. I don't feel qualified to judge about the English language but the manuscript style needs improvement according to the authors guidelines.
5. Abstracts and Introduction does give a concise information about objective of this work. Why is it needed, what novel is in this research, which papers concerned these research questions to the greenery in the Great Britain, and why? The reasons to study the chosen area in well explained? Why greenery changes in Great Britain region are important (besides urbanization)? Which studies are for this region? Partially, these questions were answered, and in my opinion, it is enough and quite efficiently.
6. Data source and processing section is completed. Authors can add more information about the datasets acquired (if it feels necessary, software used, processing methods applied to the data: which one is the final resolution as the research was conducted with datasets at different spatial resolutions? A reference is needed for the different indices.
7. Description of methodology is concise. Maybe, the classification technique could be visualized or/and quantified based on more example?

Recommendation. Summarize my comments, Overall, the manuscript is written well. I would recommend to Authors to address my comments into manuscript taking into account most of issues including motivation, methods, results analysis. These are the major revisions and after the complete elaboration, the manuscript could be accepted in this Journal.
