# Supplementary material for: Urban centre green metrics in Great Britain: A geospatial and socioecological study
Source: PLoS One. 2022 Nov 23;17(11):e0276962. doi: 10.1371/journal.pone.0276962 (PMC9683550; doi:10.1371/journal.pone.0276962)
Supplement: Attachment — Submitted filename: Response to Reviewers.docx [file pone.0276962.s006.docx]

**Submitted to *PLOS ONE***

Urban centre green metrics in Great Britain: A geospatial and socioecological study

Jake M. Robinson^*^, Suzanne Mavoa, Kate Robinson, and Paul Brindley

**Editor/reviewer feedback and author responses.**

**Date:** 07-10-2022

| **Comment** | **Response** |
| --- | --- |
| **Editor** |  |
|  | Thanks again for considering our manuscript for *PLOS ONE.* We are pleased that you and the reviewers all see the value of our manuscript.  My co-authors and I are also pleased that we addressed the major revisions and that the reviewers feel the manuscript is now ready for publication. We have also addressed the minor revisions from the latest round of feedback and agree that the reviews greatly improved the manuscript. We hope you now find it in an acceptable form for publication in *PLOS ONE.* |
| **Reviewer 1** |  |
| Minor issues  1.There are a lot of repetitive keywords (e.g., urban green infrastructure and green infrastructure).  2.The data was collected in 2019, so how did the authors draw a conclusion related to the COVID-19 pandemic? | Thanks for your feedback. To address this, we have removed some of the repetitive keywords in particular “green infrastructure”; for instance, at Lines 91, 94, 361, 363, 377, 378, 410, 552.  Thanks. We have taken your feedback on board and have removed one of the two references to COVID-19 in the discussion (at Line 555). The pandemic is a topical and a highly relevant to current urban centres and future green infrastructure management. Therefore, we left one reference to COVID-19 in the manuscript (as a relevant but not central discussion point), at Line 398: “Urban centres in Great Britain are changing, and retail outlets are closing, mainly due to the evolution of digital shopping technologies. This has been accentuated by the COVID-19 pandemic, which has devasted the traditional retail property sector”. |
| **Reviewer #3** |  |
| Now it can be considered for publication however the quality of the figures is still an issue. Authors stated they increased the dpi however, some figures become even worse (please check the pdf of your submission). Maybe it is the problem that images are exported in small dimensions (height x width in terms of pixel size). nevertheless this is a technical issue that maybe journal guide Authors. | We are pleased you feel the paper is now ready for publication and appreciate you spending time to review it.  Thanks for pointing the image issue out. Perhaps it is a conversion issue; we will double check the quality and upload new versions. We will also liaise with the journal editors to make sure they are at the appropriate resolution/quality to publish. |
